# Real Customization or Just Marketing: Are Customized Versions of Generative AI Useful?

**DOI:** 10.12688/f1000research.153129.3

**Published:** 2024-10-17

**Authors:** Eduardo C. Garrido-Merchán, Jose Luis Arroyo-Barrigüete, Francisco Borrás-Pala, Leandro Escobar-Torres, Carlos Martínez de Ibarreta, Jose María Ortíz-Lozano, Antonio Rua-Vieites

**Affiliations:** 1 Universidad Pontificia Comillas, Madrid, Community of Madrid, Spain; 2 Santalucía Chair of Analytics for Education, Madid, Spain, Spain

**Keywords:** Artificial Intelligence, ChatGPT, customisation, virtual instructor, higher education, statistics

## Abstract

**Background:**

Large Language Models (LLMs), as in the case of OpenAI ^TM^ ChatGPT-4 ^TM^ Turbo, are revolutionizing several industries, including higher education. In this context, LLMs can be personalised through customization process to meet the student demands on every particular subject, like statistics. Recently, OpenAI launched the possibility of customizing their model with a natural language web interface, enabling the creation of customised GPT versions deliberately conditioned to meet the demands of a specific task.

**Methods:**

This preliminary research aims to assess the potential of the customised GPTs. After developing a Business Statistics Virtual Professor (BSVP), tailored for students at the Universidad Pontificia Comillas, its behaviour was evaluated and compared with that of ChatGPT-4 Turbo. Firstly, each professor collected 15-30 genuine student questions from “Statistics and Probability” and “Business Statistics” courses across seven degrees, primarily from second-year courses. These questions, often ambiguous and imprecise, were posed to ChatGPT-4 Turbo and BSVP, with their initial responses recorded without follow-ups. In the third stage, professors blindly evaluated the responses on a 0-10 scale, considering quality, depth, and personalization. Finally, a statistical comparison of the systems’ performance was conducted.

**Results:**

The results lead to several conclusions. Firstly, a substantial modification in the style of communication was observed. Following the instructions it was trained with, BSVP responded in a more relatable and friendly tone, even incorporating a few minor jokes. Secondly, when explicitly asked for something like, “I would like to practice a programming exercise similar to those in R practice 4,” BSVP could provide a far superior response. Lastly, regarding overall performance, quality, depth, and alignment with the specific content of the course, no statistically significant differences were observed in the responses between BSVP and ChatGPT-4 Turbo.

**Conclusions:**

It appears that customised assistants trained with prompts present advantages as virtual aids for students, yet they do not constitute a substantial improvement over ChatGPT-4 Turbo.

## Introduction

The rapid advancements in statistical generative artificial intelligence (AI) ( Murphy, 2023), particularly in the realm of natural language processing and generation with the emergence of Large Language Models (LLMs) ( Gozalo-Brizuela and Garrido-Merchán, 2023b, Zhao et al., 2023), based on the transformers architecture, have given birth to a new paradigm in a plethora of sectors ( Gozalo-Brizuela and Garrido-Merchán, 2023a), like marketing ( Fraiwan and Khasawneh, 2023), higher education ( Baskara, 2023; Sullivan et al., 2023) and research ( Garrido-Merchán, 2023). Among the most notable developments in this field is OpenAI’s ChatGPT-4 Turbo ( OpenAI, 2023), a sophisticated language model that has demonstrated remarkable capabilities in generating human-like text ( Garrido-Merchán et al., 2023) and performing several tasks accurately ( Peng et al., 2023). This technology’s potential in the educational sector, especially in creating virtual teaching assistants ( Baidoo-Anu and Ansah, 2023), is immense. However, when customised for specific educational purposes, these AI models’ effectiveness and practical utility remain burgeoning research areas.

Customised generative AI, particularly in LLMs like ChatGPT-4, involves configuring the model with specific data or prompts for tailored tasks, such as being a virtual instructor. This conditioning enhances its effectiveness in specialised roles, like serving as a virtual professor. OpenAI’s new natural language interface for customization makes this process accessible across various fields. The relevance of this research stems from the growing demand for personalised learning in higher education. Customised AI models promise more engaging and personalised interactions, potentially transforming education. However, the true impact of these models on learning outcomes requires rigorous investigation to validate their effectiveness beyond marketing claims.

This study, therefore, focuses on evaluating the efficacy of a customised GPT version of ChatGPT-4 Turbo, developed as a Business Statistics Virtual Professor (BSVP), specifically for statistics students at the Business Faculty of Universidad Pontificia Comillas. By comparing the performance of this tailored model with the standard ChatGPT-4 Turbo in this particular task, this research aims to provide insights into the actual benefits and limitations of AI customisation in an educational context.

### Related work

The integration, challenges and opportunities of Generative AI into higher education, especially in the context of teaching, have garnered considerable attention in recent years ( Michel-Villarreal et al., 2023). This section reviews the latest research in the field ( Lo, 2023), emphasising studies that explore the role of generative AI in teaching, its application as a virtual assistant, and its contribution to academic research.

Recent studies in this domain have focused on the efficacy of generative AI in enhancing teaching methodologies ( Baidoo-Anu and Ansah, 2023). These works highlight the potential of AI in personalising learning experiences, providing real-time feedback, and augmenting traditional teaching practices ( Kasneci et al., 2023; Zhai, 2022). For example, ChatGPT has been proven helpful for lifelong learning ( Rawas, 2023), as, for instance, it can readapt the teaching lessons to the latest advances of rapidly changing technologies.

However, generative AI has also raised a debate about evaluation methodologies of higher education ( Anders, 2023), as students can use its content generation to cheat easily ( Cotton et al., 2024). For example, evaluations done by professors have changed to adapt to this paradigm shift as, for instance, traditional assessments are easier to cheat than ever with generated content of Generative AI ( Rudolph et al., 2023).

Another significant area of research involves using generative AI as virtual assistants in educational settings ( Chheang et al., 2023). These studies explore the capabilities of AI assistants in managing student inquiries, offering personalised tutoring, and facilitating learning outside the traditional classroom environment ( Ruiz-Rojas et al., 2023).

Finally, the role of generative AI in academic research ( Xames and Shefa, 2023) has been an area of growing interest ( Rahman and Watanobe, 2023). These investigations delve into how AI can assist in data analysis, brainstorming of ideas, literature review, synthetic data generation, text simplification and even in helping to write some sections of research papers, thereby augmenting the research capabilities of scholars and students alike ( Garrido-Merchán, 2023).

### Generative Pretrained Transformers (GPTs)

The evolution of Generative Pretrained Transformers (GPTs) ( Radford et al., 2018) has produced a paradigm shift in the democratisation of natural language processing (NLP) ( Chowdhary and Chowdhary, 2020). The journey began with the original GPT model ( Radford et al., 2018), introduced by OpenAI, whose novelty includes unsupervised learning to predict the next word in a sentence, not only supervised learning as was done before. More concretely, GPT’s methodology encompassed a dual-phase process: an initial ‘pre-training’ stage using an unsupervised generative approach to establish baseline parameters through language modelling, followed by a customization stage, where these parameters were refined and tailored to a specific task in a supervised, discriminative manner.

**Figure 1.**
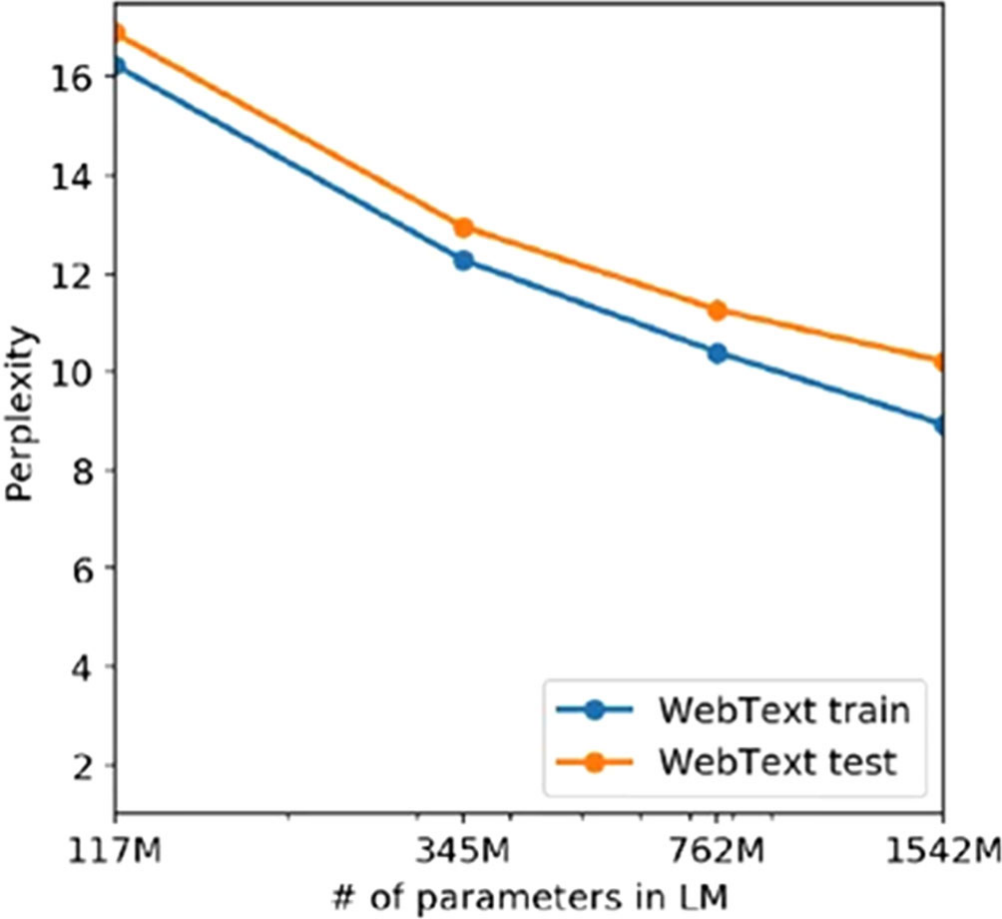
Training and test set perplexities as a function of the millions of parameters of the GPT models (Source: Radford et al., 2019).

This model laid the groundwork for more advanced iterations. GPT-2, developed by OpenAI ( Radford et al., 2019) marked a significant leap with its 1.5 billion parameters and more engineering tricks, demonstrating enhanced text generation capabilities and enabling the hypothesis that scale was all that natural language processing needs. However, its behaviour showed clues of underfitting, being its capacity, despite its 1.5 billion parameters, which were too simple for the complexity of the Webtext corpus with 45 millions of webpages, as we illustrate in Figure 1.

**Figure 2.**
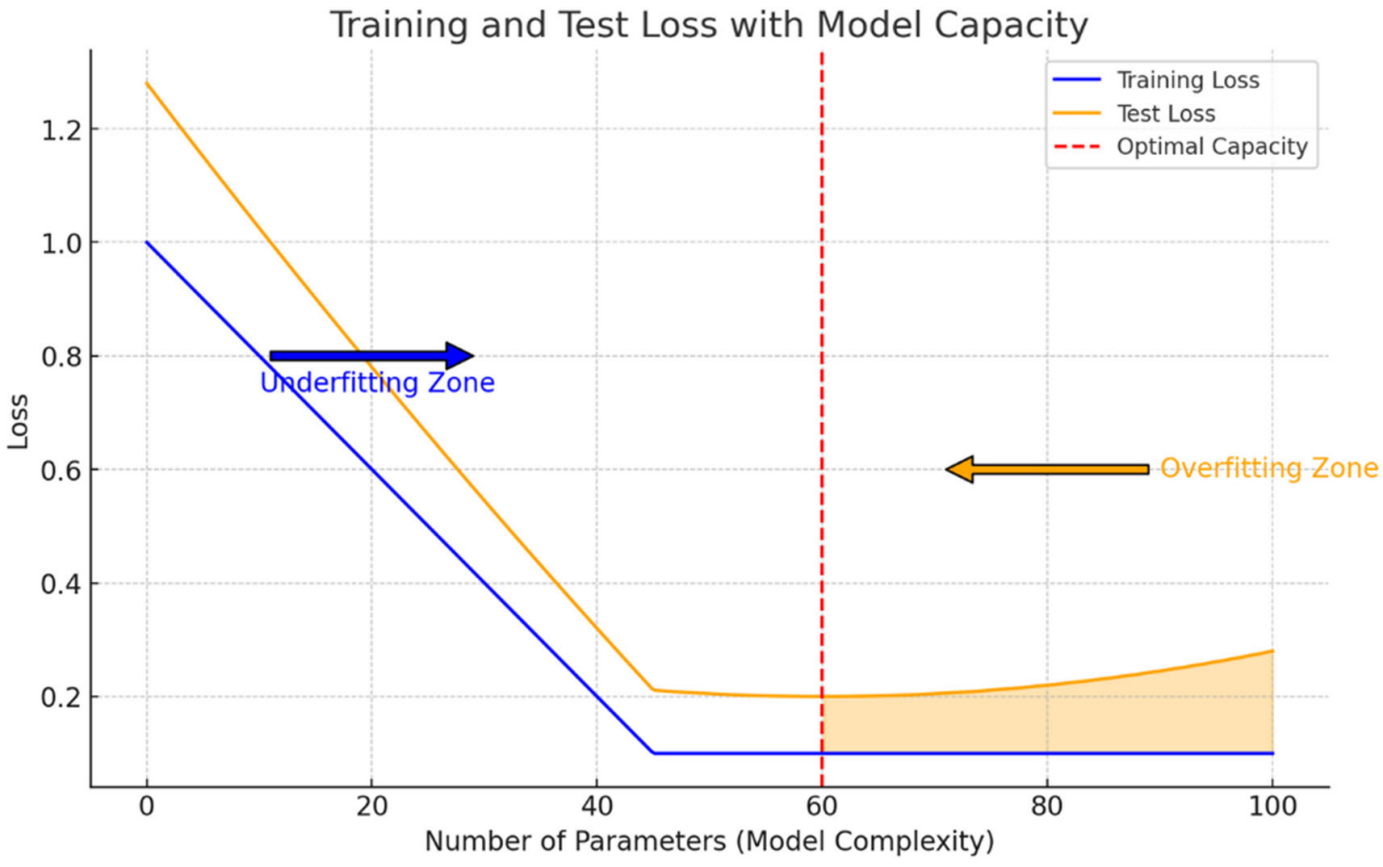
Underfitting and overfitting zones of a machine learning model with respect to its number of parameters given a dataset illustrated by the estimation of a particular loss function error in both datasets. The underfiting issue appears when the model capacity is not able to represent the complexity of the data, incurring in a higher error than the one that can be obtained by increasing the number of parameters of the model, which was what happened with GPT models with respect to the WebText dataset (Source: own elaboration).

We can clearly see how, according to this curve, the model is still in the underfitting regime. Recall that the overfitting regime starts when the test loss function curve surpasses the training set curve, indicating that the model is representing patterns that do not generalize outside of the sample which has been trained on. In order to reach the optimum point shown on Figure 2, a higher number of parameters was needed. This is the reason why GPT-3 and GPT-4 use a higher number of parameters, to try and reach this optimum point with respect to the WebText dataset, which is now more complex that in GPT-2 times and hence the model is going to require a higher capacity, because if it does not have the necessary capacity it will incur again in the underfitting issue that we have presented. We illustrate in Figure 2 an explanation of the underfitting zone suffered by the GPT-2 model and diagnosed by OpenAI researchers.

Motivated by this underfitting hypothesis, OpenAI launched GPT-3 ( Brown et al., 2020), revolutionising the field with its 175 billion parameters and offering unprecedented language understanding and generation proficiency. It is important to emphasise that each iteration of GPT has built upon the transformer architecture ( Vaswani et al., 2017). This architecture abandoned the recurrent layers used in previous models, relying instead on a self-attention mechanism that allowed the model to weigh the significance of different parts of the input data.

ChatGPT then emerged as a GPT 3.5 version that optimised the conversational experience with a user, being ChatGPT-4 ( OpenAI, 2023) and ChatGPT-4 Turbo, standing out with its enhanced capabilities and efficiency, in comparison with GPT-3 ( Peng et al., 2023). This version maintains the core transformer architecture but introduces several optimisations for speed and performance.

A critical component in developing GPT models, especially ChatGPT-4 Turbo that explains its outstanding behaviour is Reinforcement Learning from Human Feedback (RLHF) ( Christiano et al., 2017). This training approach involves fine-tuning models based on feedback from human trainers. Initially, the model generates responses based on its pretraining; these responses are then evaluated by humans who provide ratings or improved versions of the responses. The model is subsequently retrained to prefer the human-approved responses. This method ensures that the model’s outputs align more closely with human preferences, leading to more accurate and contextually appropriate responses that, now with the fine-tuned versions of ChatGPT like BSVP ( Garrido-Merchán et al., 2024b), can gain even more importance.

The fine-tuning process in GPT models allows for the customisation of the base model to suit specific applications or domains. The fine-tuning process involves training the pre-existing model on a smaller, domain-specific dataset, enabling it to adapt its responses to the nuances of a particular field or user requirement. Fine-tuning can significantly enhance the model’s performance in specialised tasks by adjusting its outputs to be more aligned with the specific content, style, or tone required by the application. This is precisely one of the advantages of its use in education.

The recent systematic review by Dong et al. (2024) highlights that research on the use of LLMs in education reveals both significant risks, such as obstruct the development of students’ critical thinking skills or lead problems in academic integrity, as well as potential positive impacts on the learning process. Some authors argue that “Large language models, such as ChatGPT, have the potential to revolutionize teaching and assist in teaching processes. […]. [For example] teachers can use large language models to create personalized learning experiences for their students” ( Kasneci et al., 2023, p. 2). Specifically addressing their use as virtual assistants in higher education, several studies suggest that LLMs can support learning ( Laato et al., 2023), contributing to personalized learning and knowledge access ( Salem & Shaalan, 2024; Yigci et al., 2024). In fact, some research already indicates significant student use of these types of virtual assistants ( Flores Limo et al., 2023). There are even proposals for chatbots specifically designed for higher education ( Wang et al., 2023), which seem to perform better than ChatGPT on tasks related to course-specific content or less commonly known topics. This is precisely what we aim to assess, by comparing the standard version of ChatGPT with its customized version.

## Methods

Initially, a virtual assistant for Statistics courses taught at Universidad Pontificia Comillas was created. The assistant was instructed via prompt with specific directions regarding communication style. The decision was made to customize the model exclusively through prompt engineering, motivated by the intent to evaluate this new personalization feature offered by OpenAI. Prompt engineering allows for the adaptation of advanced language models such as ChatGPT without the need for complex technical interventions, thereby facilitating their use by individuals without specialized programming knowledge. This method promises to democratize the creation of personalized virtual assistants, making them accessible to a broader audience. Hence, there is a keen interest in assessing its effectiveness.

Additionally, contextual documentation was provided: two books written by three professors of the subject and signatories of this research ( Borrás-Pala et al., 2019a, 2019b), as well as the R programming practices document, prepared by another three different professors, who are also authors of this work. Over three days, two authors tested the system, progressively refining the prompt until they achieved a version they considered acceptable.

The prompt utilized was designed to focus the model on key areas such as descriptive statistics, probability, and statistical inference. The prompt was structured with three priorities. The first priority was to ensure that responses were personalized, aligning with the way the subject matter is taught in the “Statistics and Probability” and “Business Statistics” courses at the Business Faculty of Universidad Pontificia Comillas. To achieve this, the model was instructed to always prioritize the content from the contextual documentation, with specific directives included. For instance, the instructions stated, “If asked about content related to descriptive statistics, probability, or inference, give absolute priority to the contents of the statistics books uploaded. Give them maximum weight and do not use other sources unless the prompt asks for content that is not contained in the books,” and “If a student asks about a practice, consult the ‘Programming Practices’ document to respond. No other source is acceptable. Only that one.” The second priority was the use of language appropriate for the average student at this university. Instructions incorporated for this purpose included “Use Spanish from Spain (Castilian),” “Do not digress, be concise,” and “When you want to say ’assume,’ use ‘suppose.’” The third priority of the prompt was to employ a communication style that is engaging and relatable to the students. This was achieved by incorporating directives such as “Adopt the tone of an influencer who popularizes content. For example, be slightly enthusiastic in your responses, using emoticons in your explanations,” and “At some point in your response, make a joke about the ICADE professor [...] to enhance the student’s experience.” These structured instructions were crafted to guide the model effectively, ensuring that its outputs were both academically aligned with the university’s standards and engaging for the students, thereby optimizing the educational interaction.

Once the system was refined, the evaluation began. The study was conducted through the assessment of BSVP’s response quality by the five professors who signed this work but did not participate in the generation and subsequent adjustment of the prompt. Specifically, the work was carried out in four different stages. Firstly, each professor collected between 15 and 30 questions posed by students of the ‘Statistics and Probability’ and ‘Business Statistics’ courses, which are taught across seven different degrees. A final sample of 136 questions was obtained. In most cases, these were second-year courses (mostly students aged 19-20) and, in some instances, third-year courses (mostly students aged 20-21). All questions had to be genuine inquiries made by students during classes or tutoring sessions. This is a highly relevant aspect, as students often struggle to clearly and precisely articulate their doubts (e.g., ‘I don’t understand what this Student’s t is about’; ‘In the Poisson binomial, how is lambda calculated?’): it’s essential to evaluate the system’s ability to respond to these kinds of questions competently, even if the formulation of the question itself is imprecise or even incorrect. If BSVP is to act as a virtual assistant for students, it should be able to answer such questions despite their ambiguity, lack of definition or even errors in the question itself. The questions collected are those that students typically ask in class or during tutoring sessions (not specifically for this study) and have been used anonymously. Intentionally, the questions collected by the professors were not coordinated, which implies that a few questions collected by one researcher might be similar to those collected by another. This occurred in some cases with questions that are very common among students. For example: ‘I don’t fully understand the difference between the intersection of two random events and one being conditioned on the other. ‘or ‘How can I tell if a problem is asking for the probability of an intersection or a conditioned event? ‘In any case, since these were real questions, the wording was never identical, allowing for the testing of both systems’ (ChatGPT-4 Turbo and BSVP) ability to respond to different formulations. In the second stage, each question was posed to ChatGPT-4 Turbo and BSVP ( Garrido-Merchán et al., 2024b), noting down both complete responses. To ensure comparability, there were no follow-up questions or clarifications; the first provided response was copied, whether satisfactory or not. In the third phase, the professors who had not participated in generating and adjusting the prompt evaluated the responses from ChatGPT-4 Turbo and BSVP, scoring them on a scale of 0 to 10. The choice of this specific scale responds to the characteristics of the Spanish university system, where it is the default scale used to evaluate university students. Therefore, the professors responsible for this evaluation are familiar with this scale. It is important to note that the evaluation was blind, as each professor assessed both responses without knowing who the author was (ChatGPT-4 Turbo or BSVP). Only the two professors who did not participate in the evaluation had this information. Specifically, three different dimensions were evaluated: quality of the response (clarity, conciseness, etc.); depth of the response (to what extent it is as complete as possible); and personalisation (degree of closeness to the way the subject is taught at the university where the study was conducted). To give a concrete example regarding the dimension of personalization, when teaching probability calculations for the normal distribution, most statistics textbooks rely on tables or statistical software. However, at our university, we emphasize that students develop the ability to perform approximate calculations without using tables or software, leveraging the properties of the normal distribution. That is, by knowing the percentage of data within the intervals of μ ± σ, 2σ, and 3σ, students should be able to estimate approximate probabilities. This is a distinction that BSVP should consider. Results are available at Garrido-Merchán et al. (2024a). Finally, in the fourth stage, a statistical comparison of the results obtained by both systems was carried out. Specifically, a paired samples t-test assuming equal variances was conducted for the mean differences in each of the three indicated dimensions.

## Results

**Figure 3.**
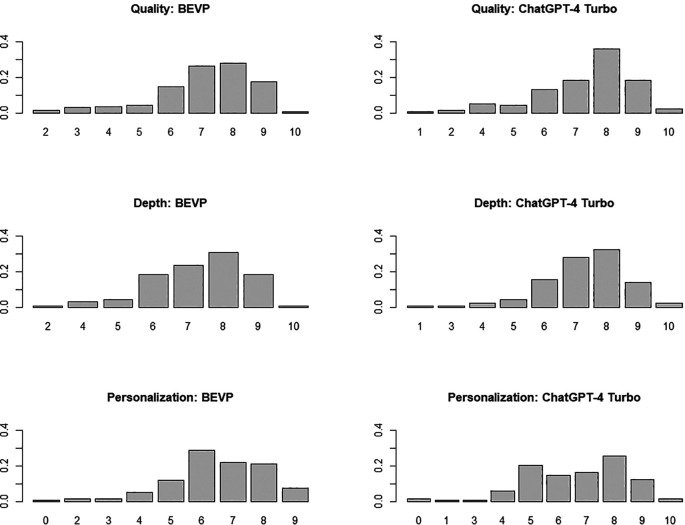
Bar plot of the scores obtained by BSVP and ChatGPT-4 Turbo in each of the three dimensions analysed (figure generated with R).

Starting with a qualitative assessment, a substantial modification in the communication style was observed. As per its training, BSVP responded in a much more approachable and friendly tone. In fact, it often began responses with phrases like ‘Dear ICADE student, …’ ^1^ ‘This question you ask is very interesting,’ or ‘Excellent question, my dear ICADE student!’ The farewells were also more cordial (‘a big hug,’ ‘I hope this has helped you’), and occasionally, they incorporated small jokes (‘Perhaps your ICADE teacher might say something different, though I doubt it. But after all, they are human, and I am not, so I know much more than them’) ^2^. Greater conciseness in the responses was also generally observed, as instructed in the training prompt. A highly relevant aspect is that when explicitly asked for something like ‘I would like to practice a programming exercise similar to those in R programming practice 3,’ BSVP was capable of providing a much superior response: having access to contextual documentation, it was able to address the request, something that was not possible for ChatGPT-4 Turbo ^3^. However, as a trade-off, the response times were generally longer. Regarding the content, a total of 136 questions were obtained, which, as mentioned, were evaluated according to three dimensions: quality, depth, and personalisation. Figure 3 shows the corresponding bar plots.

The comparative analysis of the performance of both systems (see Table 1) suggests no significant differences in any dimension: the p-values obtained across the three dimensions, all of which exceed 0.05, suggest that the observed differences may simply be due to random variations in the data sample rather than a systematic effect of the customization implemented in the BSVP. In fact, after applying the Bonferroni correction for multiple comparisons, the results become even clearer, with p-values of 0.82, 1, and 1 for Quality, Depth, and Personalisation, respectively. Additionally, to complement these results, the confidence intervals (CI) and the effect size (Cohen’s d) has been calculated for each dimension (effsize). Once again, a negligible effect size is confirmed in all cases, significantly below the threshold of 0.2 typically used to denote small effects. These effect sizes reinforce the conclusion that the customization of the BSVP system has not resulted in significant improvements in student interaction compared to the standard ChatGPT-4 Turbo model. The most interesting aspect is the absence of differences in personalisation (the degree of closeness to the way the subject is taught at the university where the study was conducted), indicating that the contextual documentation has not served to offer adapted content. As mentioned, this documentation is handy when the question explicitly references course content (i.e., ‘I would like to practice a programming exercise similar to those in R programming practice 3’), as it allows BSVP to respond competently. However, in more general questions like those included in this evaluation, which do not require the consultation of contextual documentation, there are no differences between BSVP and ChatGPT-4 Turbo.

**Table 1.** Results obtained in each dimension. Mean, standard deviation (sd), and t-test for mean difference.

	Quality	Depth	Personalisation
**BSVP: mean (sd)**	7.12 (1.60)	7.30 (1.36)	6.50 (1.57)
**ChatGPT-4 Turbo: mean (sd)**	7.30 (1.62)	7.29 (1.39)	6.64 (1.84)
**t-test**	t = -1.098 df = 135 p-value = 0.274 CI: [-0.51, 0.15] effsize = -0.11	t = 0.096 df = 135 p-value = 0.924 CI : [-0.29, 0.32] effsize = 0.01	t = -0.855 df = 135 p-value = 0.394 CI: [-0.46, 0.18] effsize = -0.08

## Discussion

The main conclusions of this research can be summarised in three key ideas. Firstly, differences in communication style are indeed noticeable. Training via prompt has created a virtual assistant whose style is distinct from that of ChatGPT-4 Turbo. Secondly, BSVP has a significant advantage over ChatGPT-4 Turbo: its contextual documentation allows it to respond to specific course content queries, which ChatGPT-4 Turbo cannot do. This is not a minor aspect, as students often pose questions this way (e.g., ‘Could you provide an example of a problem like those in chapter 4?’; ‘I don’t understand the first part of the R programming practice 6’). Lastly, regarding general content, no significant differences are evident. That is, ChatGPT-4 Turbo can answer any query like BSVP. However, we must consider that we are dealing with a subject that is quite basic and for which there is an enormous amount of information. Therefore, the responses cannot vary much in terms of quality and depth. Customisation via prompt seems to show specific improvements, especially if students prefer a friendlier communication style and targeted content queries. However, BSVP provides no benefit to students seeking doubt resolution over ChatGPT-4 Turbo.

On the other hand, as it is illustrated on the results section, the customized GPT version has shown a better performance in communication style with respect to the not customized version, which represents a critical advantage for users. The answer style provided by the standard GPT model may not be the usual way to communicate with students in different cultures, organizations and universities, depending on factors such as countries, different studies or beliefs. It is well known that university students typically interact between themselves in a specific manner ( Gorsky et al., 2006), so this style can be introduced in the configuration prompt, making the model generate text in this fashion and not sound weird by students, which is a necessity for them ( Jochim & Lenz-Kesekamp, 2024). By personalizing the model’s communication style to align with that of the professor or the expectations of the organization or university, we can enhance the benefits that generative AI models provide to students ( Tai & Chen, 2024). This personalization helps remove cultural barriers that students might face regarding the style of the texts generated by the model.

Regarding a potential improvement of performance by the BSVP customized GPT version with respect to the GPT-4 Turbo model, we do not empirically observe such behavior. Consequently, we hypothesize several causes that could be simultaneously affecting the behaviour of the BSVP customized GPT version. First, undergraduate business statistics is a subject with little dissent, in the sense that its syllabus is objective and very popular on the internet. Hence, our added specific theoretical materials of the subject do not add a significant amount of new knowledge to the GPT representation of information of its corpus encoded in its parameters. Observe that we are only describing here the theoretical content because, in the case of the practical content, if we do specific practices not done by the rest of the universities, then the customized GPT version can effectively provide unique answers as the result of its customization. We also hypothesize that if we had a subject with different schools of thought, such in the case of philosophy, for example, then, the performance of customized GPT models for education could be dramatic, as the customized GPT would be able to provide only the required answer for the subject that studies a particular school of thought. For example, if we are teaching a class about philosophy of mind, we could provide answers of both materialist or dualist beliefs by uploading files describing the schools a priori in the customization. Undoubtedly, regarding performance, the usefulness of customized GPT models in these cases would be superior than in the case of frequentist statistics.

Another critical advantage of the customized GPT versions with respect to the standard model is its usability and speed of use by the students. Instead of having to upload the subject materials to the model, students are provided with a customized version of the model that already contains the relevant subject materials. This version includes specific instructions on how to use the materials effectively, which have been carried out by the subject professor.

Hence, students are more likely to use this chatbot compared to one without the preloaded materials, as they will trust its content more, knowing that a professor has customized it. Additionally, the convenience of not needing to upload extra materials further increases its appeal. Recall that trusting generative AI is one of the issues of these systems that needs to be solved if chatbots are going to be widely used in education ( Amoozadeh et al., 2024). Moreover, the student can use the chatbot to generate personalized problems similar to those in the subject, increasing trust in the tool. The student has greater confidence that these exercises are relevant for exam preparation, rather than being general problems that may not align with the course content. Furthermore, the generated exercises can vary in difficulty, effectively assisting students in mastering challenging concepts where existing exercises may be too complex to solve without further support. These exercises can be either analytical or coding-based, providing valuable help to non-STEM students, such as those in business programs, in overcoming the challenges of STEM subjects ( Coe et al., 2008), such as statistics, particularly through practice in the R programming language.

In summary, our findings suggest that customizing AI assistants like BSVP may offer practical benefits for educators and educational institutions by enhancing student interaction through communication styles tailored to their cultural and academic expectations, saving instructors time by addressing frequent queries, and increasing student trust in a tool customized with course content. Moreover, in complex subjects or those with multiple theoretical approaches, customization can provide more precise and relevant responses, further enhancing its utility in educational settings.

The study’s main limitation is its preliminary nature. To validate our findings, an experiment where students, as end-users of BSVP, assess both systems’ responses is necessary. However, accurately assessing responses poses challenges; students probably would not be able to discriminate based on the veracity of the result: they might prefer brief answers over more accurate, complex ones; and could be influenced by the communication style, potentially skewing their judgements. Despite these obstacles, with well-designed experiments, we can further explore system differences from a student perspective and extend the research to more specialised, advanced subjects, which is what we propose as future lines of research. Another interesting research line would be to investigate whether, in subjects with different schools of thought (such as philosophy) the performance of customized GPT models is indeed substantially better than that of the general GPT, as we suspect might be the case.

### Ethical considerations

This work does not require approval from the ethics committee. The questions collected are those that students typically ask in class or during tutoring sessions (not specifically for this study) and have been used anonymously. Therefore, their consent is not required. Additionally, all professors who evaluated the quality of the responses are co-authors of this work and thus give their consent. In conclusion, approval from the ethics committee is not required. Ethical approval and participant consent were not applicable due to the nature of the study.

## Author contributions

Eduardo C. Garrido Merchán and Jose Luis Arroyo-Barrigüete contributed to the study conception and design. All authors performed material preparation, data collection, and analysis. The first draft of the manuscript was written by Eduardo C. Garrido Merchán and Jose Luis Arroyo-Barrigüete, and all authors commented on previous versions of the manuscript. All authors read and approved the final manuscript.

## Data Availability

Figshare: Real Customization or Just Marketing: Are Customized Versions of Generative AI Useful?, https://doi.org/10.6084/m9.figshare.26039461.v1 ( Arroyo-Barrigüete et al., 2024a). The project contains the following underlying data:
• Data.xlsx. Rating on a scale from 0 to 10 of all responses evaluated according to the three considered dimensions (quality, depth, and personalization). Figshare: Sample of provided responses: Real Customization or Just Marketing: Are Customized Versions of Generative AI Useful?, 10.6084/m9.figshare.26965354.v1 ( Arroyo-Barrigüete, 2024).
• This document includes a sample of responses supplied by BSVP and ChatGPT-4 Turbo. The Excel document indicates which of the responses (A or B) was provided by each of the two systems. Data are available under the terms of the Creative Commons Attribution 4.0 International license (CC-BY 4.0). A preprint of the article can be found at https://arxiv.org/abs/2312.03728. Garrido-Merchán, E. C., Arroyo-Barrigüete, J. L., Borrás-Pala, F., Escobar-Torres, L., de Ibarreta, C. M., Ortiz-Lozano, J. M., & Rua-Vieites, A. (2023). Real Customization or Just Marketing: Are Customized Versions of Chat GPT Useful?. arXiv preprint arXiv:2312.03728. Figshare: Questionary: Real Customization or Just Marketing: Are Customized Versions of Generative AI Useful?, https://doi.org/10.6084/m9.figshare.26128669.v1 ( Arroyo-Barrigüete et al., 2024b). Data are available under the terms of the Creative Commons Attribution 4.0 International license (CC-BY 4.0).

## Footnotes

^1^
ICADE - Instituto Católico de Administración y Dirección de Empresas (Catholic Institute of Business Administration and Management). It is the name of the business school of the Universidad Pontificia Comillas, where the study was conducted.

^2^
To ensure that the evaluation was blind, all these phrases were removed from the responses, so that the evaluators were not aware of them.

^3^
Logically, questions of this nature were not included in the evaluation.
