# Supplementary material for: Real Customization or Just Marketing: Are Customized Versions of Generative AI Useful?
Source: F1000Res. 2024 Oct 17;13:791. Originally published 2024 Jul 11. [Version 3] doi: 10.12688/f1000research.153129.3 (PMC11447677; doi:10.12688/f1000research.153129.3)
Supplement: Peer review history

# Reviewer report: Reviewer response for version 3

**DOI:** 10.5256/f1000research.173379.r362590

**Published:** 2025-02-19

**Authors:** Cherifa Boudia

**Affiliations:** 1 Université de Mascara, Mascara, Algeria

**Recommendations for Improvement:**

**1. Strengthen Practical Implications:**

- The paper could expand on how customization may be more beneficial in fields where factual accuracy is less rigid (e.g., philosophy, ethics, and social sciences). The authors should consider adding examples or case studies in such domains to provide more robust justification for their findings.

**2. User-Centric Evaluation:**

- Future studies may incorporate student feedback to assess user preference for customized AI assistants. A survey or qualitative analysis could provide insights into whether students perceive customization as beneficial beyond stylistic differences.

**3. Longitudinal Study:**

- A long-term evaluation of BSVP’s impact on student learning outcomes would provide additional insights into its educational value. The authors should discuss the feasibility of conducting a follow-up study to track performance over time.

**4. Clarify Customization Process Further:**

- While the study provides details on BSVP’s customization, additional technical insights into the specific modifications made to the model’s prompt structure and its effect on response generation could further enhance replicability.

**5. Broader Statistical Analysis:**

- The study currently employs t-tests and confidence intervals, which are appropriate. However, considering additional statistical techniques such as regression analysis or ANOVA could offer a more nuanced understanding of differences between BSVP and ChatGPT-4 Turbo.

**6. Discussion of Limitations and Bias:**

- The paper does discuss limitations, but a deeper reflection on potential biases in question selection and evaluation scoring would improve transparency. For example, how might the experience level of the evaluating professors influence scoring consistency?

**Final Verdict:**

 The study is well-conducted, methodologically sound, and presents a valuable contribution to AI in education. The findings suggest that while AI customization enhances user experience, it does not necessarily improve factual accuracy or depth of knowledge delivery. The work is suitable for indexing and contributes meaningful insights into the effectiveness of AI customization in education. Addressing the above recommendations would further enhance its scientific rigor and practical relevance.

Is the work clearly and accurately presented and does it cite the current literature?

Yes

If applicable, is the statistical analysis and its interpretation appropriate?

Yes

Are all the source data underlying the results available to ensure full reproducibility?

Yes

Is the study design appropriate and is the work technically sound?

Yes

Are the conclusions drawn adequately supported by the results?

Yes

Are sufficient details of methods and analysis provided to allow replication by others?

Yes

Reviewer Expertise:

eduation, e-learning, teaching and learning stategies,

I confirm that I have read this submission and believe that I have an appropriate level of expertise to confirm that it is of an acceptable scientific standard, however I have significant reservations, as outlined above.

# Reviewer report: Reviewer response for version 3

**DOI:** 10.5256/f1000research.173379.r332743

**Published:** 2024-10-21

**Authors:** FX. Risang Baskara

**Affiliations:** 1 English Letters Department, Universitas Sanata Dharma, Depox, Indonesia

Dear Authors,

 Thank you for the opportunity to review the revised version of your manuscript. I have carefully examined the changes made in response to previous reviewer comments and the overall quality of the paper. This report provides a comprehensive assessment of the revised manuscript.

 1. Overview

 The study presents a timely and relevant investigation into the effectiveness of customized versions of large language models (LLMs) in educational settings, specifically comparing a standard ChatGPT-4 Turbo model with a customized Business Statistics Virtual Professor (BSVP). The research question is well-defined, and the methodology is generally sound. The revisions have significantly improved the paper's clarity, depth, and scientific rigor.

 2. Literature Review

 The authors have successfully addressed the previous concern regarding the literature review. The inclusion of more recent and relevant studies on AI in education (e.g., Dong et al., 2024; Flores Limo et al., 2023; Laato et al., 2023) provides a stronger foundation for the research and better contextualizes the work within the rapidly evolving field.

 3. Methodology

 The methodology section has been substantially improved:

 a) BSVP Customization: The detailed explanation of the prompt engineering process and the specific instructions used for customization greatly enhances the reproducibility of the study.

 b) Evaluation Criteria: The clarification on the use of the 0-10 scale and how professors were instructed to assess responses is helpful.

 c) Sample Size: The justification for the sample size of 136 questions is now adequately explained.

 4. Statistical Analysis

 The statistical analysis has been significantly enhanced:

 a) Effect Sizes: The inclusion of Cohen's d for each dimension provides a clearer understanding of the magnitude of differences.

 b) Confidence Intervals: The addition of confidence intervals in Table 1 improves the interpretation of results.

 c) Multiple Comparisons: The application of the Bonferroni correction for multiple comparisons strengthens the validity of the statistical analysis.

 5. Results and Discussion

 The expanded discussion section offers a more nuanced interpretation of the results:

 a) Practical Implications: The authors have provided a thorough exploration of the practical implications for educators and educational institutions.

 b) Limitations: The discussion of study limitations, particularly regarding the generalizability from a single subject area, is well-articulated.

 c) Future Research: The proposed directions for future research, especially the suggestion to investigate customized GPT models in subjects with different schools of thought, are valuable.

 6. Data Availability

 The addition of sample responses from both BSVP and ChatGPT-4 Turbo enhances the reproducibility of the study.

 Minor Points

- The explanation of the underfitting issue in GPT models is clear and well-illustrated.
- The rationale for not using BLEU and ROUGE metrics is well-argued and appreciated.

7. Conclusion

 The revised manuscript represents a significant improvement over the previous version. The authors have been responsive to reviewer feedback and have made appropriate revisions to address all major concerns. The study now provides a more comprehensive and rigorous analysis of the potential and limitations of customized AI models in educational settings.

 The revised paper also makes a valuable contribution to the field of AI in education. It is well-written, methodologically sound, and provides important insights into the effectiveness of customized LLMs in educational contexts. I believe it is now suitable for indexing and will be of interest to researchers and practitioners in the field of educational technology and AI.

 Thank you for the opportunity to review this interesting and timely research.

Is the work clearly and accurately presented and does it cite the current literature?

Partly

If applicable, is the statistical analysis and its interpretation appropriate?

Partly

Are all the source data underlying the results available to ensure full reproducibility?

Yes

Is the study design appropriate and is the work technically sound?

Yes

Are the conclusions drawn adequately supported by the results?

Partly

Are sufficient details of methods and analysis provided to allow replication by others?

Partly

Reviewer Expertise:

Technology-Enhanced Language Learning (TELL)visibiliyBlended Learning Approaches in EFL ContextsvisibilitFlipped Classroom Methodologies for Language AcquisitionvisibilitArtificial Intelligence Applications in Language EducationvisibilitComputer-Assisted Language Learning (CALL)visibilitDigital Tools and Platforms for EFL TeachingvisibiliyInnovative Pedagogies in Second Language Acquisition

I confirm that I have read this submission and believe that I have an appropriate level of expertise to confirm that it is of an acceptable scientific standard.

# Reviewer report: Reviewer response for version 2

**DOI:** 10.5256/f1000research.172088.r326147

**Published:** 2024-10-04

**Authors:** Erik Carbajal-Degante

**Affiliations:** 1 Universidad Nacional Autonoma de Mexico, Mexico City, Mexico City, Mexico

I appreciate the authors' willingness to address the suggestions. I believe their work has significantly improved with the changes made. I give my approval for indexing of this article.

Is the work clearly and accurately presented and does it cite the current literature?

Partly

If applicable, is the statistical analysis and its interpretation appropriate?

Partly

Are all the source data underlying the results available to ensure full reproducibility?

Partly

Is the study design appropriate and is the work technically sound?

Yes

Are the conclusions drawn adequately supported by the results?

Yes

Are sufficient details of methods and analysis provided to allow replication by others?

Partly

Reviewer Expertise:

Artificial intelligence, computer vision, natural language processing.

I confirm that I have read this submission and believe that I have an appropriate level of expertise to confirm that it is of an acceptable scientific standard.

# Reviewer report: Reviewer response for version 2

**DOI:** 10.5256/f1000research.172088.r326146

**Published:** 2024-10-01

**Authors:** María Beatriz Corchuelo Martínez-Azua

**Affiliations:** 1 Department of Economics, Universidad de Extremadura,, Badajoz, Extremadura, Spain

The authors have considered the recommendations made and the current version of the article has been significantly improved. It is considered suitable for indexing.

Is the work clearly and accurately presented and does it cite the current literature?

Partly

If applicable, is the statistical analysis and its interpretation appropriate?

Yes

Are all the source data underlying the results available to ensure full reproducibility?

Partly

Is the study design appropriate and is the work technically sound?

Yes

Are the conclusions drawn adequately supported by the results?

Yes

Are sufficient details of methods and analysis provided to allow replication by others?

Yes

Reviewer Expertise:

Economics, Teaching Innovation

I confirm that I have read this submission and believe that I have an appropriate level of expertise to confirm that it is of an acceptable scientific standard.

# Reviewer report: Reviewer response for version 2

**DOI:** 10.5256/f1000research.172088.r326478

**Published:** 2024-09-26

**Authors:** FX. Risang Baskara

**Affiliations:** 1 English Letters Department, Universitas Sanata Dharma, Depox, Indonesia

Summary of the Article: This study investigates the effectiveness of customized versions of large language models (LLMs) in educational settings, specifically comparing a standard ChatGPT-4 Turbo model with a customized Business Statistics Virtual Professor (BSVP) designed for university students. The research aims to assess whether customized AI models offer significant advantages over general-purpose models in addressing student queries in a specific subject area. The study collected 136 genuine student questions from statistics courses, had both systems respond to these questions, and then had professors blindly evaluate the responses on quality, depth, and personalization. The results showed no statistically significant differences between the two systems, leading to the conclusion that while customized assistants may have some advantages, they do not constitute a substantial improvement over ChatGPT-4 Turbo for the tested scenario.

 Detailed Responses to Review Questions:

1. Is the work clearly and accurately presented and does it cite the current literature? Answer: Partly

The paper is generally well-structured and presents the research clearly. However, there are areas for improvement:

 a) Literature Review: While the paper cites relevant literature, the review could be more comprehensive, particularly regarding recent developments in AI for education. The authors should expand their literature review to include more recent studies on LLMs in educational contexts, especially those discussing customization efforts similar to their own.

 b) Clarity of Presentation: Some sections, particularly the methodology and results, could benefit from more detailed explanations. For instance, the process of customizing the BSVP could be described more thoroughly.

 c) Current Literature: The paper would benefit from including more up-to-date references on the use of AI in education, particularly studies published in the last 1-2 years, to better contextualize their work within the rapidly evolving field.

 Recommendation: The authors should expand their literature review, incorporating more recent studies on AI in education. They should also provide more detailed explanations in the methodology and results sections.

1. Is the study design appropriate and is the work technically sound? Answer: Yes

The overall study design is appropriate for addressing the research question. The use of genuine student questions and blind evaluation by professors are strengths of the methodology. However, there are areas where the technical soundness could be improved:

 a) Sample Size: The authors should justify the choice of 136 questions or acknowledge it as a potential limitation if it's considered small for robust statistical analysis.

 b) Evaluation Criteria: While the use of a 0-10 scale for evaluation is explained, more detail on how professors were instructed to apply this scale would be beneficial.

 c) Customization Process: More technical details on how BSVP was customized, including specific prompts or fine-tuning methods used, would enhance the study's reproducibility.

 Recommendation: The authors should provide more details on their sample size justification, evaluation criteria instructions, and the technical aspects of the BSVP customization process.

1. Are sufficient details of methods and analysis provided to allow replication by others? Answer: Partly

The paper provides a good overview of the methodology, but some crucial details are missing that would be necessary for full replication:

 a) BSVP Customization: More specific information on how BSVP was customized, including the exact prompts or instructions used, would be essential for replication.

 b) Evaluation Process: While the evaluation process is described, more details on how professors were instructed to assess the responses would be helpful.

 c) Statistical Analysis: The statistical methods are described briefly, but more details on the specific tests used and any data preprocessing steps would aid replication.

 Recommendation: The authors should provide a more detailed description of the BSVP customization process, including specific prompts used. They should also elaborate on the instructions given to professors for evaluation and provide more details on their statistical analysis methods.

1. If applicable, is the statistical analysis and its interpretation appropriate? Answer: Partly

The statistical analysis, while appropriate, is relatively basic and could be expanded:

 a) Effect Size: In addition to p-values, the authors should consider reporting effect sizes to give a better sense of the magnitude of any differences.

 b) Confidence Intervals: Including confidence intervals would provide more information about the precision of the estimates.

 c) Multiple Comparisons: If multiple t-tests were performed, the authors should consider adjusting for multiple comparisons.

 d) Interpretation: The interpretation of the statistical results could be more nuanced, discussing not just the lack of significant differences but also what the results suggest about the practical implications of customization.

 Recommendation: The authors should expand their statistical analysis to include effect sizes and confidence intervals. They should also consider adjusting for multiple comparisons if applicable and provide a more nuanced interpretation of their results.

1. Are all the source data underlying the results available to ensure full reproducibility? Answer: Yes

The authors have made their data available on Figshare, which is commendable for ensuring reproducibility. However, to further enhance reproducibility:

 a) Data Description: A more detailed description of the dataset, including variable definitions and any data cleaning steps, would be helpful.

 b) Code Availability: If any custom code was used for analysis, making this available would further aid reproducibility.

 Recommendation: While the data is available, the authors should provide a more detailed description of the dataset and consider making any custom analysis code available.

1. Are the conclusions drawn adequately supported by the results? Answer: Partly

The main conclusions are supported by the results, but there are areas where the discussion and conclusions could be strengthened:

 a) Implications: The authors could provide a more in-depth discussion of the implications of their findings for the field of AI in education.

 b) Limitations: A more thorough discussion of the study's limitations, including the generalizability of results from a single subject area, would strengthen the paper.

 c) Future Research: More specific suggestions for future research directions based on their findings would be valuable.

 d) Practical Applications: The authors could expand on the practical implications of their findings for educators and educational institutions considering the use of customized AI assistants.

 Recommendation: The authors should expand their discussion section to more thoroughly explore the implications and limitations of their findings. They should also provide more specific recommendations for future research and discuss practical applications of their results.

 Conclusion: This paper addresses an important and timely topic in the field of AI and education. While it provides valuable insights, there are several areas where the methodology, analysis, and discussion could be strengthened. The most critical points that must be addressed to make the article scientifically sound are:

1. Expanding the literature review to include more recent, relevant studies.
2. Providing more detailed information on the BSVP customization process.
3. Enhancing the statistical analysis with effect sizes and confidence intervals.
4. Offering a more nuanced interpretation of the results and their implications.

By addressing these points, along with the other suggestions provided, the authors can significantly improve the scientific rigor and impact of their study.

Is the work clearly and accurately presented and does it cite the current literature?

Partly

If applicable, is the statistical analysis and its interpretation appropriate?

Partly

Are all the source data underlying the results available to ensure full reproducibility?

Yes

Is the study design appropriate and is the work technically sound?

Yes

Are the conclusions drawn adequately supported by the results?

Partly

Are sufficient details of methods and analysis provided to allow replication by others?

Partly

Reviewer Expertise:

Technology-Enhanced Language Learning (TELL)visibiliyBlended Learning Approaches in EFL ContextsvisibilitFlipped Classroom Methodologies for Language AcquisitionvisibilitArtificial Intelligence Applications in Language EducationvisibilitComputer-Assisted Language Learning (CALL)visibilitDigital Tools and Platforms for EFL TeachingvisibiliyInnovative Pedagogies in Second Language Acquisition

I confirm that I have read this submission and believe that I have an appropriate level of expertise to confirm that it is of an acceptable scientific standard, however I have significant reservations, as outlined above.

# Response

**Published:** 2024-10-11

**Authors:** Jose Luis Arroyo-Barrigüete

**Affiliations:** Quantitative Methods, Universidad Pontificia Comillas Facultad de Ciencias Economicas y Empresariales, Madrid, Community of Madrid, Spain

Detailed Responses to Review Questions:

**Is the work clearly and accurately presented and does it cite the current literature?**

 Answer: Partly

 The paper is generally well-structured and presents the research clearly. However, there are areas for improvement:

 a) Literature Review: While the paper cites relevant literature, the review could be more comprehensive, particularly regarding recent developments in AI for education. The authors should expand their literature review to include more recent studies on LLMs in educational contexts, especially those discussing customization efforts similar to their own.

 b) Clarity of Presentation: Some sections, particularly the methodology and results, could benefit from more detailed explanations. For instance, the process of customizing the BSVP could be described more thoroughly.

 c) Current Literature: The paper would benefit from including more up-to-date references on the use of AI in education, particularly studies published in the last 1-2 years, to better contextualize their work within the rapidly evolving field.

**Recommendation:** The authors should expand their literature review, incorporating more recent studies on AI in education. They should also provide more detailed explanations in the methodology and results sections.

**Response: **We appreciate the reviewer's meticulous evaluation of our paper. We have noticed that, although the journal's website indicates that this review pertains to version 2 of the manuscript, it actually refers to the initial version (version 1). This is likely due to the close timing between when we uploaded the new version and when the review was conducted.

 The new version (version 2) incorporated the comments from the first two reviewers. Consequently, many of the issues mentioned (which were indeed weaknesses of the initial version) have already been addressed. For example, the need for a more extensive and recent literature review (points a and c) and a better explanation of the methodological and results sections (point b) have been addressed. Both aspects were also mentioned by the other two reviewers and have been incorporated into the version 2. You can find a detailed explanation of these improvements in our responses to the first two reviewers.

 However, as described below, some of the reviewer's comments had not been previously suggested. Therefore, we have incorporated them into the new version (version 3) that we are submitting along with this response letter.

**Recommendation: Is the study design appropriate and is the work technically sound?**

 Answer: Yes

 The overall study design is appropriate for addressing the research question. The use of genuine student questions and blind evaluation by professors are strengths of the methodology. However, there are areas where the technical soundness could be improved:

 a) Sample Size: The authors should justify the choice of 136 questions or acknowledge it as a potential limitation if it's considered small for robust statistical analysis.

**Response: **We conducted an initial sample size estimation aiming for a power of 0.8, a p-value of 0.05, and to detect an effect size of 0.25 in a two-tailed paired t-test. This resulted in a required sample of 128 questions. However, we decided to evaluate 136 questions to account for the possibility of encountering issues that might lead to discarding some data. Ultimately, all questions were valid, so our final sample consisted of 136 questions.

**Recommendation: **b) Evaluation Criteria: While the use of a 0-10 scale for evaluation is explained, more detail on how professors were instructed to apply this scale would be beneficial.

**Response: **The reason for this is the grading system used in Spanish universities. In this system, both assignments and exams are evaluated on a scale from 0 to 10: scores from 0 to 4.99 are failing grades, 5 to 6.99 are passing grades, 7 to 8.99 are considered "good," and 9 to 10 are "excellent." Consequently, university professors are comfortable using this scoring scale because it is the scale they use when evaluating students. We included this explanation in version 2: “The choice of this specific scale responds to the characteristics of the Spanish university system, where it is the default scale used to evaluate university students. Therefore, the professors responsible for this evaluation are familiar with this scale.”

**Recommendation: **c) Customization Process: More technical details on how BSVP was customized, including specific prompts or fine-tuning methods used, would enhance the study's reproducibility.

**Response: **This comment was also raised previously, and we addressed it in version 2 by adding the following explanation:

 “ *The decision was made to customize the model exclusively through prompt engineering, motivated by the intent to evaluate this new personalization feature offered by OpenAI. Prompt engineering allows for the adaptation of advanced language models such as ChatGPT without the need for complex technical interventions, thereby facilitating their use by individuals without specialized programming knowledge. This method promises to democratize the creation of personalized virtual assistants, making them accessible to a broader audience. Hence, there is a keen interest in assessing its effectiveness.*

*[…]*

*The prompt utilized was designed to focus the model on key areas such as descriptive statistics, probability, and statistical inference. The prompt was structured with three priorities. The first priority was to ensure that responses were personalized, aligning with the way the subject matter is taught in the "Statistics and Probability" and "Business Statistics" courses at the Business Faculty of Universidad Pontificia Comillas. To achieve this, the model was instructed to always prioritize the content from the contextual documentation, with specific directives included. For instance, the instructions stated, "If asked about content related to descriptive statistics, probability, or inference, give absolute priority to the contents of the statistics books uploaded. Give them maximum weight and do not use other sources unless the prompt asks for content that is not contained in the books," and "If a student asks about a practice, consult the 'Programming Practices' document to respond. No other source is acceptable. Only that one." The second priority was the use of language appropriate for the average student at this university. Instructions incorporated for this purpose included "Use Spanish from Spain (Castilian)," "Do not digress, be concise," and "When you want to say 'assume,' use 'suppose.'" The third priority of the prompt was to employ a communication style that is engaging and relatable to the students. This was achieved by incorporating directives such as "Adopt the tone of an influencer who popularizes content. For example, be slightly enthusiastic in your responses, using emoticons in your explanations," and "At some point in your response, make a joke about the ICADE professor [...] to enhance the student's experience." These structured instructions were crafted to guide the model effectively, ensuring that its outputs were both academically aligned with the university's standards and engaging for the students, thereby optimizing the educational interaction.”*

**Recommendation: Are sufficient details of methods and analysis provided to allow replication by others?**

 Answer: Partly

 The paper provides a good overview of the methodology, but some crucial details are missing that would be necessary for full replication:

 a) BSVP Customization: More specific information on how BSVP was customized, including the exact prompts or instructions used, would be essential for replication.

**Response: **As noted earlier, we included a more detailed explanation on this matter in version 2.

**Recommendation: **b) Evaluation Process: While the evaluation process is described, more details on how professors were instructed to assess the responses would be helpful.

**Response: **The instructors were directed to assess the responses as follows. For evaluating the dimensions of quality (such as clarity and conciseness) and depth (the extent to which the response is as complete as possible), they were instructed to use the same criteria they would apply when grading student work. Regarding the dimension of personalization (the degree to which the response aligns with how the subject is taught at the university where the study was conducted), they were to assess whether the response was consistent with the specific content and teaching methods used at Universidad Pontificia Comillas.

 To illustrate with a concrete example, when teaching probability calculations for the normal distribution, most statistics textbooks rely on tables or statistical software. However, at our university, we emphasize that students develop the ability to perform approximate calculations without using tables or software, leveraging the properties of the normal distribution. That is, by knowing the percentage of data within the intervals of μ ± σ, 2σ, and 3σ, students should be able to estimate approximate probabilities. This is a distinction that BSVP should consider; when addressing a question related to the normal distribution, it should provide a response aligned with this method of calculation. The same applies to other topics that have slight differences compared to how they are presented in other statistics textbooks. We have added a clarification on this matter in version 3.

**Recommendation: **c) Statistical Analysis: The statistical methods are described briefly, but more details on the specific tests used and any data preprocessing steps would aid replication.

**Response: **Since the evaluation was conducted by the authors themselves, data preprocessing was unnecessary; the scores assigned to the responses were error-free. However, regarding the methodology used, we have included an additional clarification to the paper. We specified that the paired samples t-tests were performed assuming equal variances because, in all three cases, Levene's test confirmed that there were no statistically significant differences between the variances of the two groups (p-value > 0.05).

**Recommendation: If applicable, is the statistical analysis and its interpretation appropriate?**

 Answer: Partly

 The statistical analysis, while appropriate, is relatively basic and could be expanded:

 a) Effect Size: In addition to p-values, the authors should consider reporting effect sizes to give a better sense of the magnitude of any differences.

**Response: **We agree that including effect sizes is important. We have already incorporated this into version 2 (see Table 1).

**Recommendation: **b) Confidence Intervals: Including confidence intervals would provide more information about the precision of the estimates.

**Response: **We have included the confidence intervals in Table 1.

**Recommendation: **c) Multiple Comparisons: If multiple t-tests were performed, the authors should consider adjusting for multiple comparisons.

**Response: **We completely agree with this point. We did not include it in the previous version because, since no significant differences were found, we considered the correction unnecessary. However, we have now incorporated it into version 3 by applying a Bonferroni correction to the p-values obtained.

**Recommendation: **d) Interpretation: The interpretation of the statistical results could be more nuanced, discussing not just the lack of significant differences but also what the results suggest about the practical implications of customization.

**Response: **We acknowledge that this was a weakness in the initial version. However, in version 2, the reviewer can observe that the discussion section has been substantially expanded.

**Recommendation: Are all the source data underlying the results available to ensure full reproducibility?**

 Answer: Yes

 The authors have made their data available on Figshare, which is commendable for ensuring reproducibility. However, to further enhance reproducibility:

 a) Data Description: A more detailed description of the dataset, including variable definitions and any data cleaning steps, would be helpful.

**Response: **In version 2, we have already included additional information about the data used in the Figshare repository, as requested by one of the initial reviewers.

**Recommendation: **b) Code Availability: If any custom code was used for analysis, making this available would further aid reproducibility.

**Response: **The statistical analysis is actually quite simple and can be performed using any statistical software. We used R simply because it is the standard tool in our research, not because complex calculations were necessary. The computations are indeed straightforward. For example, the command to calculate a t-test is as follows:

 t.test(PEE_Personalization, GPT_Personalization, var.equal = TRUE, paired = TRUE)

**Recommendation: Are the conclusions drawn adequately supported by the results?**

 Answer: Partly

 The main conclusions are supported by the results, but there are areas where the discussion and conclusions could be strengthened:

 a) Implications: The authors could provide a more in-depth discussion of the implications of their findings for the field of AI in education.

**Response: **As previously mentioned, in version 2 the discussion section was substantially expanded, which includes a more in-depth discussion of the implications of our findings.

**Recommendation: **b) Limitations: A more thorough discussion of the study's limitations, including the generalizability of results from a single subject area, would strengthen the paper.

**Response: **We completely agree with this comment. For that reason, in version 2 we specifically addressed this point. Our opinion is that if we had a subject with different schools of thought, such in the case of philosophy, for example, then, the performance of customized GPT models for education could be dramatic, as the customized GPT would be able to provide only the required answer for the subject that studies a particular school of thought. For example, if we are teaching a class about philosophy of mind, we could provide answers of both materialist or dualist beliefs by uploading files describing the schools a priori in the customization. Undoubtedly, regarding performance, the usefulness of customized GPT models in these cases would be superior than in the case of frequentist statistics.

**Recommendation: **c) Future Research: More specific suggestions for future research directions based on their findings would be valuable.

**Response: **We have incorporated a new future line of research related to the previous point: “Another interesting research line would be to investigate whether, in subjects with different schools of thought (such as philosophy) the performance of customized GPT models is indeed substantially better than that of the general GPT, as we suspect might be the case.”

**Recommendation: **d) Practical Applications: The authors could expand on the practical implications of their findings for educators and educational institutions considering the use of customized AI assistants.

**Response: **We have included an additional paragraph on this matter: “In summary, our findings suggest that customizing AI assistants like BSVP may offer practical benefits for educators and educational institutions by enhancing student interaction through communication styles tailored to their cultural and academic expectations, saving instructors time by addressing frequent queries, and increasing student trust in a tool customized with course content. Moreover, in complex subjects or those with multiple theoretical approaches, customization can provide more precise and relevant responses, further enhancing its utility in educational settings.”

**Recommendation: **Conclusion: This paper addresses an important and timely topic in the field of AI and education. While it provides valuable insights, there are several areas where the methodology, analysis, and discussion could be strengthened. The most critical points that must be addressed to make the article scientifically sound are:

 Expanding the literature review to include more recent, relevant studies.

 Providing more detailed information on the BSVP customization process.

 Enhancing the statistical analysis with effect sizes and confidence intervals.

 Offering a more nuanced interpretation of the results and their implications.

 By addressing these points, along with the other suggestions provided, the authors can significantly improve the scientific rigor and impact of their study.

**Response: **We appreciate the reviewer's insightful comments and suggestions. We believe that the new version of our manuscript has addressed the weaknesses highlighted

# Reviewer report: Reviewer response for version 1

**DOI:** 10.5256/f1000research.167979.r317573

**Published:** 2024-09-04

**Authors:** María Beatriz Corchuelo Martínez-Azua

**Affiliations:** 1 Department of Economics, Universidad de Extremadura,, Badajoz, Extremadura, Spain

Thank you for the opportunity to review the paper "Real Customization or Just Marketing: Are Customized Versions of Generative AI Useful?". This research examines the effectiveness of generative language models, focusing on ChatGPT, in education. It compares ChatGPT with a customized assistant, the Business Statistics Virtual Professor (BSVP), designed for students at Universidad Pontificia Comillas. The study assesses the potential of these AI models to enhance learning, highlighting the need for effective personalization and fine-tuning. The findings reveal no significant differences between the two methods, suggesting that personalization may not be as impactful as anticipated. The study concludes that while personalized AI models hold promise, further research is needed to optimize their use in education.

 The following are some comments and suggestions that could enhance the overall impact of the research conducted:

 The work is presented clearly and precisely, with the study's methodology, results, and implications detailed effectively. The conclusions are well-supported by the results, and the study references relevant works, including previous studies and the context of the Spanish university system, which strengthens the evaluation criteria used in the research. Howev **er,** the provided excerpts lack specific references to recent literature beyond the authors' previous work. To broaden the study's perspective, it would be beneficial to include references that discuss the use of language models in diverse educational contexts, both locally and internationally. This could offer a more comprehensive view of the applicability of the findings.

 The study commendably provides access to the source data underlying the results, with data availability on Figshare and appropriate licensing that supports transparency and reproducibility. However, the research's impact could be further enhanced by offering more detailed descriptions of the dataset, which would improve its usability for future research.

 While statistical results are presented, the study would benefit from a more in-depth discussion of their significance and practical implications. For instance, exploring how the results might affect educational practice or influence the adoption and use of virtual assistants in higher education would be valuable. Such an analysis could provide readers with a clearer understanding of the real-world applications and potential impact of the findings.

Is the work clearly and accurately presented and does it cite the current literature?

Partly

If applicable, is the statistical analysis and its interpretation appropriate?

Yes

Are all the source data underlying the results available to ensure full reproducibility?

Partly

Is the study design appropriate and is the work technically sound?

Yes

Are the conclusions drawn adequately supported by the results?

Yes

Are sufficient details of methods and analysis provided to allow replication by others?

Yes

Reviewer Expertise:

Economics, Teaching Innovation

I confirm that I have read this submission and believe that I have an appropriate level of expertise to confirm that it is of an acceptable scientific standard, however I have significant reservations, as outlined above.

# Response

**Published:** 2024-09-17

**Authors:** Jose Luis Arroyo-Barrigüete

**Affiliations:** Quantitative Methods, Universidad Pontificia Comillas Facultad de Ciencias Economicas y Empresariales, Madrid, Community of Madrid, Spain

We sincerely thank the reviewer for the time dedicated to evaluating our research. Below, we provide responses to her comments.

1. **The work is presented clearly and precisely, with the study's methodology, results, and implications detailed effectively. The conclusions are well-supported by the results, and the study references relevant works, including previous studies and the context of the Spanish university system, which strengthens the evaluation criteria used in the research. However, the provided excerpts lack specific references to recent literature beyond the authors' previous work. To broaden the study's perspective, it would be beneficial to include references that discuss the use of language models in diverse educational contexts, both locally and internationally. This could offer a more comprehensive view of the applicability of the findings.**

We sincerely appreciate your comment. Due to the space limitations required by the journal for a Brief Report, it is not possible to include a large number of new references or text. However, we have reviewed a significant number of papers on the subject and selected those that we believe provide relevant information for the present research. These have been included in the paper at the end of the “Related Work” section:

  

- Dong, B., Bai, J., Xu, T., & Zhou, Y. (2024, April). Large Language Models in Education: A Systematic Review. In 2024 6th International Conference on Computer Science and Technologies in Education (CSTE) (pp. 131-134). IEEE.
- Flores Limo, F. A.,... & Arias Gonzáles, J. L. (2023). Personalized tutoring: ChatGPT as a virtual tutor for personalized learning experiences. Przestrzeń Społeczna (Social Space), 23(1), 293-312.
- Laato, S., Morschheuser, B., Hamari, J., & Björne, J. (2023, July). AI-assisted learning with ChatGPT and large language models: Implications for higher education. In 2023 IEEE International Conference on Advanced Learning Technologies (ICALT) (pp. 226-230). IEEE.
- Salem, M., Shaalan, K. (2024). ChatGPT: Advancing Education with Virtual Assistants. In: Hassanien, A.E., Zheng, D., Zhao, Z., Fan, Z. (eds) Business Intelligence and Information Technology. BIIT 2023. Smart Innovation, Systems and Technologies, vol 394. Springer, Singapore. https://doi.org/10.1007/978-981-97-3980-6_25
- Wang, K., Ramos, J., & Lawrence, R. (2023). ChatEd: a chatbot leveraging ChatGPT for an enhanced learning experience in higher education. arXiv preprint arXiv:2401.00052.
- Yigci, D., Eryilmaz, M., Yetisen, A. K., Tasoglu, S., & Ozcan, A. (2024). Large Language Model‐Based Chatbots in Higher Education. Advanced Intelligent Systems, 2400429. https://doi.org/10.1002/aisy.202400429

**2. The study commendably provides access to the source data underlying the results, with data availability on Figshare and appropriate licensing that supports transparency and reproducibility. However, the research's impact could be further enhanced by offering more detailed descriptions of the dataset, which would improve its usability for future research.**

 In order to offer more detailed descriptions of the dataset, we have uploaded a sample of the responses provided by both BSVP and ChatGPT-4 Turbo to a public repository. We have included the link to these files in the data availability statement:

 Figshare: Sample of provided responses: Real Customization or Just Marketing: Are Customized Versions of Generative AI Useful?, https://doi.org/10.6084/m9.figshare.26965354.v1  

 This document includes a sample of responses supplied by BSVP and ChatGPT-4 Turbo.

**While statistical results are presented, the study would benefit from a more in-depth discussion of their significance and practical implications. For instance, exploring how the results might affect educational practice or influence the adoption and use of virtual assistants in higher education would be valuable. Such an analysis could provide readers with a clearer understanding of the real-world applications and potential impact of the findings.**

 After checking our document we definitely agree with this observation and feel very grateful with the reviewer to have told us to provide a wider discussion section. Please observe that we have augmented the discussion section regarding how the customized model can impact the students use of generative AI tools. In particular, we have focused our analysis in how does communication style conditions the student use of these models and how it also influences the fact that the customization has been done by a professor of the subject, clearly modifying the trust that the students feel with respect to the generative AI system once that they know that it is, somehow, validated and explored by the professors of the subject. Moreover, we have also given an argument on why, regarding statistics, the performance of the customized version does not clearly outperform the standard version, based on the fact that undergraduate statistics is a popular topic on the internet and it is knowledge accepted by all communities. We hypothesize that for another more subjective topics with different schools of thought, like for example different fields of philosophy like philosophy of mind, the personalized answers can outperform the standard answers as they will focus only on the school of thought being taught in the subject. Finally, in this discussion, we also provided some additional examples and references to support our arguments.

# Reviewer report: Reviewer response for version 1

**DOI:** 10.5256/f1000research.167979.r302467

**Published:** 2024-08-13

**Authors:** Erik Carbajal-Degante

**Affiliations:** 1 Universidad Nacional Autonoma de Mexico, Mexico City, Mexico City, Mexico

**Summary**:

 The present study conducts a comparative analysis to identify significant differences between the widely recognized Large Language Model ChatGPT and a customized GPT-based assistant, specifically designed for tailoring question-and-answer tasks for students within a particular subject at a specific university. The clarity and organization of the paper structure is good. Please, find bellow my comments:

**Comments:**

- In the section on GPTs, I recommend that the authors clarify the concept of underfitting and provide a detailed explanation of the specific indicators or evidence of underfitting they are referring to in relation to the GPTs family.
- The process of fine-tuning involves a comprehensive adjustment of the model's architecture, hyperparameters, and learning mechanisms to ensure effective assimilation of the new information during inference. In light of this, authors should expand on this topic to clarify whether a specific fine-tuning method was applied in the development of the BSVP assistant, or if the customization was limited to prompt engineering.
- In the Methods section, I recommend providing evidence of the types of instructions given via prompts for the BSVP assistant.
- In the Results section, this study could be enhanced by incorporating additional NLP metrics, such as BLEU and ROUGE, which provide reliable and complementary data alongside the expert evaluations from the professors. Including these metrics would significantly increase the paper's value by adding a quantitative dimension to the analysis.
- The statistical results indicate no significant difference between the outcomes produced by the GPT and BSVP methods. I recommend that the authors provide a more in-depth analysis of the interpretation of their results, particularly regarding the insights provided by the p-value.

In my opinion, addressing these comments would significantly improve the quality of this research, enhancing its suitability for indexing.

Is the work clearly and accurately presented and does it cite the current literature?

Partly

If applicable, is the statistical analysis and its interpretation appropriate?

Partly

Are all the source data underlying the results available to ensure full reproducibility?

Partly

Is the study design appropriate and is the work technically sound?

Yes

Are the conclusions drawn adequately supported by the results?

Yes

Are sufficient details of methods and analysis provided to allow replication by others?

Partly

Reviewer Expertise:

Artificial intelligence, computer vision, natural language processing.

I confirm that I have read this submission and believe that I have an appropriate level of expertise to confirm that it is of an acceptable scientific standard, however I have significant reservations, as outlined above.

# Response

**Published:** 2024-09-17

**Authors:** Jose Luis Arroyo-Barrigüete

**Affiliations:** Quantitative Methods, Universidad Pontificia Comillas Facultad de Ciencias Economicas y Empresariales, Madrid, Community of Madrid, Spain

First of all, we would like to thank the reviewer for their comments and the time dedicated to evaluating our work. We will now proceed to respond to the issues raised.

**In the section on GPTs, I recommend that the authors clarify the concept of underfitting and provide a detailed explanation of the specific indicators or evidence of underfitting they are referring to in relation to the GPTs family**

 We thank the reviewer for coming up with such an interesting topic. The underfitting issue was diagnosed by OpenAI scientists as the loss function of the training and test sets of GPT-2 with respect to the WebText corpus did not converge, as we illustrate in the paper in the section on GPTs. We also provided a very detailed explanation on the underfitting issue with a Figure where it can clearly be seen how the loss functions of GPT-2 were on the underfitting zone.

**The process of fine-tuning involves a comprehensive adjustment of the model's architecture, hyperparameters, and learning mechanisms to ensure effective assimilation of the new information during inference. In light of this, authors should expand on this topic to clarify whether a specific fine-tuning method was applied in the development of the BSVP assistant, or if the customization was limited to prompt engineering.**

 We have explained in more detail this issue, adding the following text:

*The decision was made to customize the model exclusively through prompt engineering, motivated by the intent to evaluate this new personalization feature offered by OpenAI. Prompt engineering allows for the adaptation of advanced language models such as ChatGPT without the need for complex technical interventions, thereby facilitating their use by individuals without specialized programming knowledge. This method promises to democratize the creation of personalized virtual assistants, making them accessible to a broader audience. Hence, there is a keen interest in assessing its effectiveness.*

**In the Methods section, I recommend providing evidence of the types of instructions given via prompts for the BSVP assistant.**

 We appreciate the comment and agree that it is important to expand on this point. Therefore, we have added the following text:

*The prompt utilized was designed to focus the model on key areas such as descriptive statistics, probability, and statistical inference. The prompt was structured with three priorities. The first priority was to ensure that responses were personalized, aligning with the way the subject matter is taught in the "Statistics and Probability" and "Business Statistics" courses at the Business Faculty of Universidad Pontificia Comillas. To achieve this, the model was instructed to always prioritize the content from the contextual documentation, with specific directives included. For instance, the instructions stated, "If asked about content related to descriptive statistics, probability, or inference, give absolute priority to the contents of the statistics books uploaded. Give them maximum weight and do not use other sources unless the prompt asks for content that is not contained in the books," and "If a student asks about a practice, consult the 'Programming Practices' document to respond. No other source is acceptable. Only that one." The second priority was the use of language appropriate for the average student at this university. Instructions incorporated for this purpose included "Use Spanish from Spain (Castilian)," "Do not digress, be concise," and "When you want to say 'assume,' use 'suppose.'" The third priority of the prompt was to employ a communication style that is engaging and relatable to the students. This was achieved by incorporating directives such as "Adopt the tone of an influencer who popularizes content. For example, be slightly enthusiastic in your responses, using emoticons in your explanations," and "At some point in your response, make a joke about the ICADE professor [...] to enhance the student's experience." These structured instructions were crafted to guide the model effectively, ensuring that its outputs were both academically aligned with the university's standards and engaging for the students, thereby optimizing the educational interaction.*

**In the Results section, this study could be enhanced by incorporating additional NLP metrics, such as BLEU and ROUGE, which provide reliable and complementary data alongside the expert evaluations from the professors. Including these metrics would significantly increase the paper's value by adding a quantitative dimension to the analysis.**

 We a priori agree with the reviewer in that providing a quantitative measure of the quality of the generated text is such an interesting idea. In fact, it is an idea that we will research in the short future. The problem is that current measures as BLEU and ROUGE only determine the syntactic similarity of the text being generated with a reference text. We provide a full explanation to the reviewer about why using BLEU and ROUGE is not the best idea in this particular case scenario. However, we feel very grateful because this comment has inspired us an exciting new research line to design better quantitative measure in the era of generative AI. Now, we provide the explanation:

 In the domain of natural language processing, traditionally, BLEU and ROUGE have been widely used metrics to assess the quality of machine-generated text by comparing it with reference texts. However, the application of these metrics in evaluating AI-generated content for technical or academic fields, such as the one covered in this paper, presents significant limitations that we illustrate in this subsection, making them a biased and not representative measure of evaluating the quality of the texts generated by AI, than for this particular scenario can only be evaluated by a expert commitee. In order to develop our argument we present an  illustrative example. Consider the following reference text:

 "Poisson Regression models are best used for modeling events where the outcomes are counts. Or, more specifically, count data: discrete data with non-negative integer values that count something, like the number of times an event occurs during a given timeframe or the number of people in line at the grocery store."

 Now, to illustrate why quantitative measures such as BLEU and ROUGE are biased and do not represent the real loss function of this problem. We generate two texts with the generative AI. One of those texts is almost identical to the reference text, but the concept is wrong, as it considers that the Poisson distribution must be used for continuous variables. The other text is significantly different from the reference text, however, the concept is correct and clearly explained, being a better text than the similar text. Critically, the first text is going to score a higher BLEU and ROUGE than the second text, which is precisely what we do not want to happen.

 Similar but wrong text: “Poisson Regression models are best suited for modeling events where the outcomes are counts, specifically continuous data with non-negative integer values following a normal distribution, like the number of times an event occurs within a specific period or the number of people in line at a store.”

 In particular, we have computed the BLEU score for this text with the following value: BLEU = 0.836.

 Different but correct text: “Poisson Regression models are ideal for predicting events represented by count variables. This type of data involves discrete, non-negative integers that quantify occurrences, such as the frequency of an event within a specific period or the number of customers queuing at a store.”

 In this case, the BLEU score is 0.117, which is much lower than the previous case. This example illustrates how these quantitative measures, although they appeal ideal for this problem, will not measure the quality of the text generated, because we are not interested on replicating the reference text but in generating a new way to correctly express the underlying idea. If the text is almost similar but the concept is wrong, then, the text is wrong. However, as we have seen, BLEU and ROUGE will score a high value. Consequently, we rely on qualitative evaluations done by the experts, that give us a better estimate of the quality of the text, as BLEU and ROUGE are not statistics necessarily correlated with the quality of the text being generated. 

**The statistical results indicate no significant difference between the outcomes produced by the GPT and BSVP methods. I recommend that the authors provide a more in-depth analysis of the interpretation of their results, particularly regarding the insights provided by the p-value.**

 The explanation of the p-value has been expanded, including the effect size (Cohen's D) for each case in table 1. This explicitly demonstrates that the effect is negligible in all instances: it is below the 0.2 threshold typically considered indicative of a small effect. The following text has been added:

*the p-values obtained across the three dimensions, all of which exceed 0.05, suggest that the observed differences may simply be due to random variations in the data sample rather than a systematic effect of the customization implemented in the BSVP. Additionally, to complement these results, the effect size (Cohen's d) has been calculated for each dimension (effsize). Once again, a negligible effect size is confirmed in all cases, significantly below the threshold of 0.2 typically used to denote small effects. These effect sizes reinforce the conclusion that the customization of the BSVP system has not resulted in significant improvements in student interaction compared to the standard ChatGPT-4 Turbo model.*

**In my opinion, addressing these comments would significantly improve the quality of this research, enhancing its suitability for indexing.**

 We sincerely appreciate the referee's comments.
